# An Efficient ABC_DE_Based Hybrid Algorithm for Protein–Ligand Docking

**DOI:** 10.3390/ijms19041181

**Published:** 2018-04-13

**Authors:** Boxin Guan, Changsheng Zhang, Yuhai Zhao

**Affiliations:** School of Computer Science and Engineering, Northeastern University, Shenyang 110819, China; neuguanboxin@163.com (B.G.); zhangchangsheng@ise.neu.edu.cn (C.Z.)

**Keywords:** drug design, protein–ligand docking, artificial bee colony, differential evolution

## Abstract

Protein–ligand docking is a process of searching for the optimal binding conformation between the receptor and the ligand. Automated docking plays an important role in drug design, and an efficient search algorithm is needed to tackle the docking problem. To tackle the protein–ligand docking problem more efficiently, An ABC_DE_based hybrid algorithm (ADHDOCK), integrating artificial bee colony (ABC) algorithm and differential evolution (DE) algorithm, is proposed in the article. ADHDOCK applies an adaptive population partition (APP) mechanism to reasonably allocate the computational resources of the population in each iteration process, which helps the novel method make better use of the advantages of ABC and DE. The experiment tested fifty protein–ligand docking problems to compare the performance of ADHDOCK, ABC, DE, Lamarckian genetic algorithm (LGA), running history information guided genetic algorithm (HIGA), and swarm optimization for highly flexible protein–ligand docking (SODOCK). The results clearly exhibit the capability of ADHDOCK toward finding the lowest energy and the smallest root-mean-square deviation (RMSD) on most of the protein–ligand docking problems with respect to the other five algorithms.

## 1. Introduction

The development of new drugs is costly and inefficient, so it is urgent to apply new theoretical methods and new technologies to improve it. Computer aided drug design (CADD) is developed gradually under the strong impetus of this social demand [1,2]. CADD takes advantage of advanced multidisciplinary technology, methods, and achievements, and has been a necessary basic tool for drug design. Its application shortens the process of drug research and reduces the cost of drug discovery. Protein–ligand docking, as an important part of CADD, is a computer simulation to predict the binding pose when the three-dimensional structures of protein receptors and ligands are known [3,4,5,6]. The purpose of protein–ligand docking is to find the conformation with the lowest energy when a ligand binds the active region of a receptor. Protein–ligand docking has been widely used in understanding molecular biological functions, predicting drug toxicity, screening virtual drugs and so on [7,8,9,10]. For the main process of protein–ligand docking, all possible active sites of the receptor are first detected. Then, the active site that binds to the ligand is determined, the position of the ligand is constantly adjusted, and different conformations are obtained according to the complementary principle of docking. The results are sorted by a specific scoring function, and the binding pose with the lowest energy score is finally found.

A scoring function [11,12,13,14,15,16] and a search algorithm [17,18,19] are the necessary tools of a docking method for solving the two goals above. The scoring function is used to evaluate the affinity between the receptor and the ligand for each conformation [20]. Scoring functions express the geometric complementarity and the energy strength of the interaction based on the physicochemical characteristics of the amino acids in contact with each other [21]. In the docking process, it is necessary to obtain the binding affinity accurately as the basis for optimization. Furthermore, the scoring function can also effectively help the docking to explore the binding space of the ligand. The scoring function can be directly used as the fitness function of the search algorithm.

The purpose of the search algorithm is to identify the optimal binding pose between the receptor and the ligand. The performance of the search algorithm directly affects the efficiency of molecular docking. The ideal search algorithm should be able to enumerate all possible binding poses between ligands and receptors, but this is difficult to achieve because the search space involved in molecular docking is huge. Many evolutionary computation methods have been presented for solving protein–ligand docking problems [22,23,24,25,26,27,28]: for example, simulated annealing (SA) [29], genetic algorithm (GA) [30], Lamarckian genetic algorithm (LGA) [31], running history information guided genetic algorithm (HIGA) [32], and swarm optimization for highly flexible protein–ligand docking (SODOCK) [33]. These methods have been applied to solve docking problems, but they have some drawbacks. As two standard evolutionary algorithms, SA and GA are not combined with local searches, so they can no longer search better solutions in the neighborhood of the current solution and the diversity of solutions cannot be maintained. LGA is a hybrid of GA and local search, HIGA is an improved algorithm based on running history information, and SODOCK is a hybrid of particle swarm optimization (PSO) and local search. Although LGA, HIGA, and SODOCK have local searches, their main disadvantage is the high probability of becoming trapped by local optima because of their single search strategy. Therefore, developing a more efficient and reliable search algorithm is necessary.

To solve the protein–ligand docking problem more efficiently, an efficient ABC_DE_based hybrid algorithm for protein–ligand docking (ADHDOCK) is proposed in the article. The novel method is a hybrid of artificial bee colony algorithm [34] and differential evolution algorithm [35]. Artificial bee colony (ABC) and differential evolution (DE) are two practical evolutionary computation methods, and they have been widely used in various applications. In ADHDOCK, these two algorithms share the same population and execute in parallel. An adaptive population partition (APP) mechanism incorporated into ADHDOCK is applied to automatically partition the population into two subpopulations and allocate them to ABC and DE. The reasonable allocation of computing resources makes the novel method make better use of the advantages of ABC and DE and prevents the single use of one algorithm from falling into the local optima.

The environment and the scoring function of AutoDock 4.2.6 are used as the experimental platform in the article [36]. AutoDock, as a free and open source molecular docking program, is one of the most commonly used docking software, and it provides great convenience for researchers to develop new algorithms on the existing program. AutoDock first forms a box by using the amino acid residues around the active site of the receptor. Subsequently, the program scans with different types of atoms as probes, calculates the energy of the grid, and searches for the ligand within the range of the box. Finally, every complex is scored according to the different conformation, orientation, and position of the ligand. To test the power of ADHDOCK, we performed an experiment on a set of protein–ligand complexes from PDBbind 2017 [37]. The performance of ADHDOCK, ABC, DE, LGA, HIGA, and SODOCK is compared in these datasets. Computer simulation results reveal that ADHDOCK is superior to the other methods regarding obtained energy and root-mean-square deviation (RMSD), success rate, convergence performance, data distribution, and hypothesis test.

## 2. Results

### 2.1. Data Preparation and Parameter Setting

Fifty X-ray crystallographic complexes are randomly chosen from PDBbind 2017 [37] to compare the capability of the different docking methods. One rigid protein receptor and one flexible ligand are prepared before docking. Through AutoDock’s file format, input files of both protein and ligand are preserved. The preparation process of proteins is as below: (1) get rid of ligands, the water molecule and metal ion not being included in binding sites; (2) restore the residues of missing atoms; (3) mix hydrogen with all atoms, integrate nonpolar hydrogen atoms, and allot partial charges; and (4) assign the parameter of solvent. The input files of the ligand are gained by the following process: (1) acquire the ligand atom coordinates from PDB files; (2) mix hydrogen with all atoms, integrate nonpolar hydrogen atoms, and allot the partial charge; and (3) define the torsions and a rigid root of the ligand. The file preparation stage of a molecule is carried out through AutoDock Tools. The degrees of freedom include three parameters denote the translation of the ligand relative to a specified center, a quaternion represents the orientation of the ligand with four parameters, and *T* torsion parameters where *T* is the number of rotatable bonds.

The semi-empirical free energy force field [13] is used as the scoring function in the experiment. The force field includes six pair-wise evaluations (*V*) and an estimate of the conformational entropy lost upon binding (Δ*S_conf_*):

(1) $$\Delta G=(V_{bound}^{L-L}-V_{unbound}^{L-L})+(V_{bound}^{P-P}-V_{unbound}^{P-P})+(V_{bound}^{P-L}-V_{unbound}^{P-L}+\Delta S_{conf})$$

where *L* represents the ligand and *P* represents the protein. Each of the pair-wise energetic terms is expressed as the sum of dispersion/repulsion in which the parameters are based on the Amber force field, hydrogen bonding, electrostatics, and desolvation.

(2) $$V=W_{vdw}\sum_{i,j}^{}\left(\frac{A_{ij}}{r_{ij}^{12}}-\frac{B_{ij}}{r_{ij}^{6}}\right)+W_{hbond}\sum_{i,j}E\left(t\right)\left(\frac{C_{ij}}{r_{ij}^{12}}-\frac{D_{ij}}{r_{ij}^{10}}\right)+W_{elec}\sum_{i,j}\frac{q_{i}q_{j}}{e\left(r_{ij}\right)r_{ij}}+W_{sol}\sum_{i,j}\left(S_{i}V_{j}+S_{j}V_{i}\right)e^{\left(-r_{ij}^{2}/2\sigma^{2}\right)}$$

The docking power of six algorithms (ADHDOCK, ABC, DE, LGA, HIGA and SODOCK) is compared. For each tested algorithm, the number of iterations was 27,000 and the number of individuals was 100. The other parameters were set in accordance with previous studies [31,32,33,34,35], and the details are shown in Table 1.

### 2.2. Comparison of Energy and Root-Mean-Square Deviation (RMSD)

**Figure 1:**
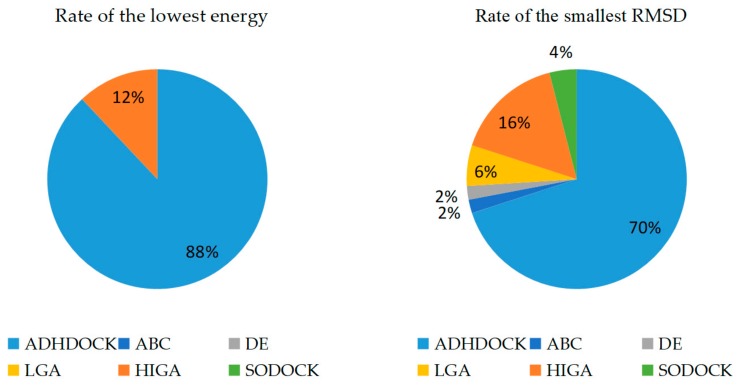
Rate of the lowest energy and the smallest root-mean-square deviation (RMSD).

The main purpose of our experiment is to find the lowest energy because the docked energy values are the most important criterion to evaluate the performance of the tested algorithms. RMSD is an important index to evaluate the protein–ligand re-docking algorithms, and it is obtained by comparing the re-docking result with the real crystallographic structure of the complex. The smaller RMSD of a docked conformation is considered to be the more accurate solution to the docking problem and the stronger search capability of the used algorithm. A docking can be considered successful if the RMSD is smaller than a given threshold 2.0 Å after docking. Each method is run thirty times independently for each protein–ligand complex. The conformation obtained from the first run is called the first predicted conformation. The best conformation with the lowest energy value obtained during all thirty runs under the condition of RMSD < 2.0 Å is called the best predicted conformation, and the docking results of RMSD ≥ 2.0 Å are not counted. The success cases, the average RMSD (all cases) and the average RMSD (RMSD < 2.0 Å) of the first predicted conformations are calculated and recorded in Table 2. The lowest energy and RMSD of the best predicted conformation are recorded, and the results are shown in Table 3. The bold fonts are used to highlight which algorithm wins in each complex. The rate of the lowest energy and the smallest RMSD found by different tested algorithms is clearly shown in Figure 1.

### 2.3. Convergence Analysis

**Figure 2:**
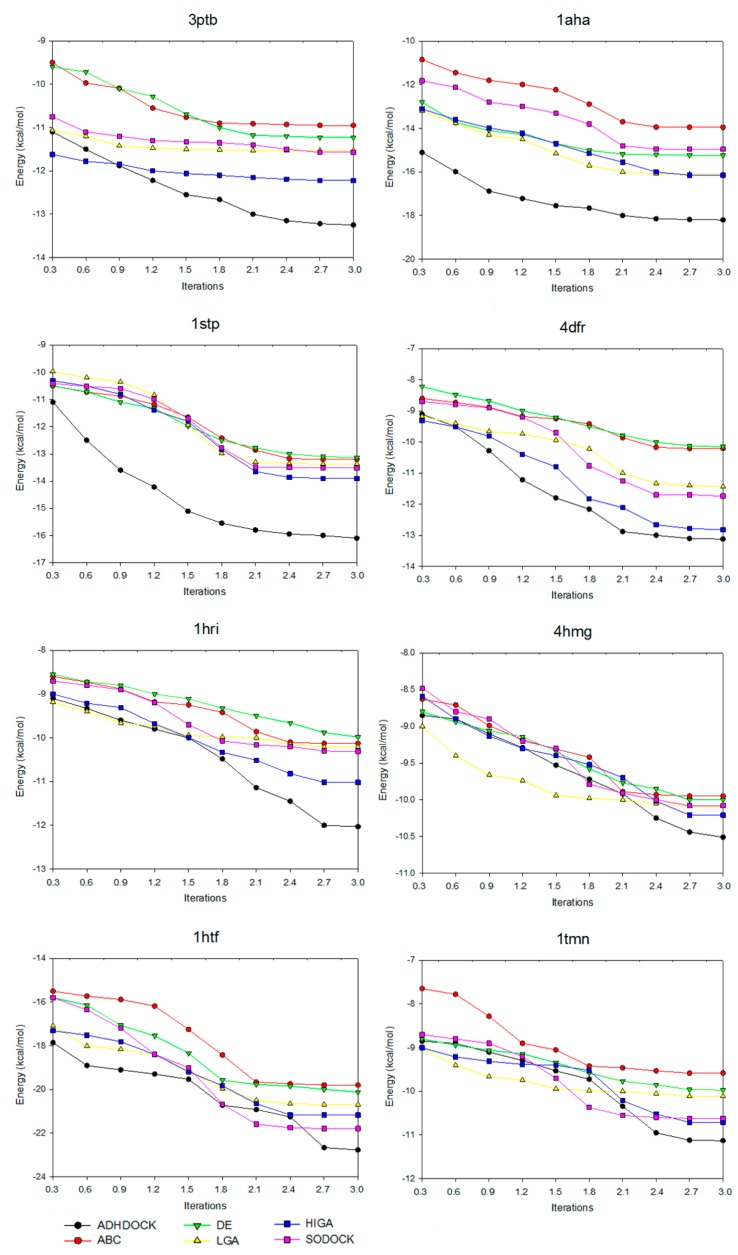
Convergence diagrams of six tested algorithms.

Convergence means that, after iterating for several times, the convergence curve of the target solution is likely to be steady. Figure 2 shows the convergence diagrams of the six tested algorithms for solving some representative complexes. The number of iterations is 3000, 6000, 9000, 12,000, 15,000, 18,000, 21,000, 24,000, 27,000 and 30,000, respectively, and the values are utilized as the horizontal axis of the convergence diagrams. Under different times of iteration, the energy value of each algorithm is referred to as the vertical axis. With the increase of the number of iterations, the power grows at the early stage of each algorithm. However, the power of certain algorithms is likely to be fixed in the later stage due to the loss of evolutionary ability and the reduction of population diversity. This phenomenon is referred to as premature convergence.

### 2.4. Data Distribution Analysis

**Figure 3:**
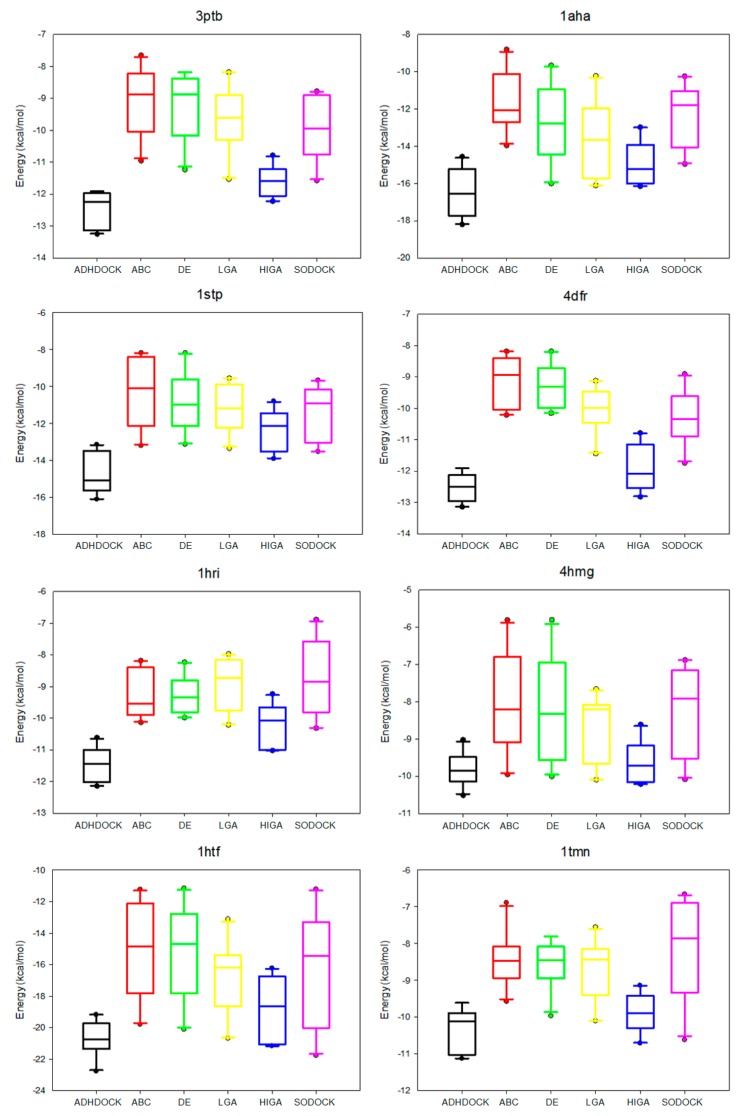
Box plots of six tested algorithms.

The data distribution can reflect the algorithm stability and the data concentration. We calculate the median, the first quartile, the third quartile, the minimum, and the maximum of the energy values of each complex, and then we apply the five statistical quantities to obtain the box plots (Figure 3). The median is suitable as a centralized trend value and not impacted by the extreme data. The median is an important measure of whether the distribution of data is dispersed or concentrated. Moreover, the first quartile is the upper boundary of the box and the third quartile is the lower boundary and the size of the box also reflects the concentration of the data. The dots outside the minimum value and maximum value are called outliers, and these outliers have a negative result on data distribution.

### 2.5. Hypothesis Test

To demonstrate whether the algorithm is more applicable to solve the protein–ligand problem, we adopt the hypothesis test with the confidence level of 0.05 in the section. Comparing Algorithm 1 with Algorithm 2, when the *p*-value between them is less than 0.05, it shows that Algorithm 1 is significantly better than Algorithm 2. For every complex, ten best values solved by each tested algorithm are taken out as experimental data. The results of the comparison of ADHDOCK with ABC, DE, LGA, HIGA and SODOCK are shown in Table 4.

## 3. Discussion

In this study, we demonstrated the superiority of ADHDOCK by many experiments. The number of success cases and the average RMSDs are recorded in Table 1. ADHDOCK finds forty-four success cases in the fifty complexes for the first predicted conformation, and the success rate of the algorithm is higher than that of ABC, DE, LGA, HIGA and SODOCK. In addition, the average RMSD (all cases) and the average RMSD (RMSD < 2.0 Å) with their respective standard deviations of ADHDOCK are the smallest compared with the other tested algorithms. It can be seen in Table 2 that ADHDOCK is well capable of finding the lowest energy values on forty-four out of fifty complexes. HIGA searches for the lowest values on six out of fifty complexes. For the smallest RMSD of the fifty complexes, ADHDOCK finds thirty-five smallest values, HIGA finds eight smallest values, LGA finds three smallest values, SODOCK finds two smallest values, and ABC and DE find one smallest value each. The experimental results indicate that ADHDOCK can solve protein–ligand docking problems more effectively and accurately than the five other comparative algorithms.

For the convergence analysis, ABC is prematurely convergent after iterating 18,000 times in 3tpb. DE is prematurely convergent after iterating 21,000 times in 1aha. The figure shows that, when compared with other algorithms on preventing premature convergence and enhancing solution quality, ADHDOCK is better. Moreover, for this case, as shown in 1aha and 1stp, it is obvious that the convergent trajectory of ADHDOCK is far away from others. For 3ptb and 4dfr, the convergent trajectories of ADHDOCK and HIGA are similar, but ADHDOCK is better than HIGA with the iteration of the optimization process. For 1hri, 4hmg, 1htf and 1tmn, ADHDOCK does not show the advantage in the early iteration, but the method gets better solutions than the other algorithms in the late iteration. From the above analyses, we can arrive at a conclusion that ADHDOCK significantly outperforms the other five comparative algorithms.

It is apparent in the box plots shown in Figure 3 that the median energy of ADHDOCK is lower than that of the other tested algorithms. The size of the boxes of ADHDOCK is the shortest in most complexes. It is evident that ADHDOCK has the most concentrated data distribution among the six test algorithms. For the outliers, ADHDOCK has no outliers, which indicates that the algorithm can reduce the randomness of the evolutionary process. It can be concluded from the above analysis that ADHDOCK is stable for protein–ligand docking.

As shown in Table 3, ADHDOCK is significantly better than other tested algorithms in thirty-nine out of fifty complexes. In 1mrg, 3hvt, 1cdg, and 1rds, ADHDOCK is significantly better than four tested algorithms. In 1phg, 1eap, and 1lic, ADHDOCK is significantly better than three tested algorithms. In 6rnt and 1hri, ADHDOCK is significantly better than two tested algorithms. In 1nco and 1hpv, ADHDOCK is significantly better than one tested algorithm. We can conclude from the results of the hypothesis tests that the performance of ADHDOCK is the best.

## 4. Materials and Methods

### 4.1. Framework of ADHDOCK

**Figure 4:**
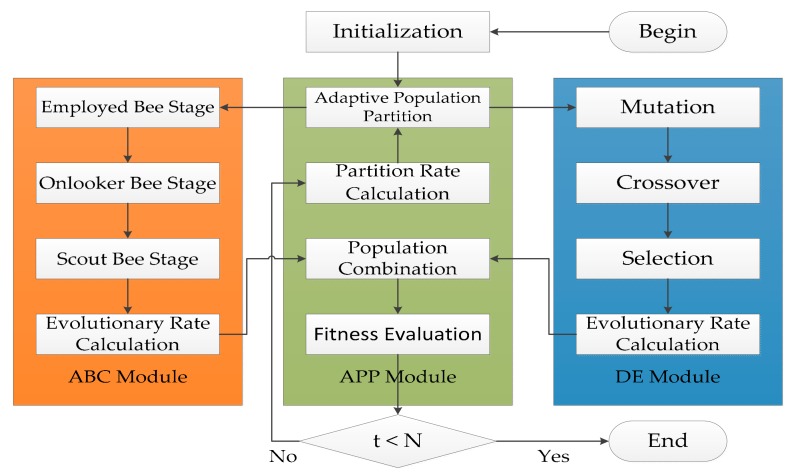
Block diagram of ADHDOCK.

ADHDOCK is a hybrid search algorithm based on ABC [34] and DE [35], and it is designed for protein–ligand docking. The block diagram of ADHDOCK is shown in Figure 4. ADHDOCK consists of three main modules. (1) ABC module: The population in the module is evolved according to ABC algorithm. (2) DE module: The population in the module is evolved by using DE algorithm. (3) Adaptive population partition (APP) module: The population is partitioned based on the partition rate. In the following sections, the three main parts are described in detail.

### 4.2. ABC Module

ABC [34] is an optimization method that simulates the foraging behavior of the bee colony. The method consists of three kinds of bees: employed bees, onlooker bees and scout bees. The solution to the problem to be optimized is considered the food source, and the richer the food, the better the quality of the solution. The employed bees correspond to the food source they collect, and they store the information about the food source and share it with other bees at a certain probability. The number of employed bees is equal to the number of food sources, as one employed bee is related to only one food source. The onlooker bees observe the dance of the employed bees in the hive to determine which food source to choose. The scout bees randomly search for new food sources near the hive. The basic structure of the ABC algorithm can be split into employed bee stage, onlooker bee stage and scout stage.

At the employed bee stage, each employed bee hunts for a new food source near the existing food source *x_i_* by

(3) $$v_{i}\left(t+1\right)=x_{i}\left(t\right)+\varphi \left(x_{i}\left(t\right)-x_{k}\left(t\right)\right)$$

where *x_k_* is a randomly selected food source; *t* is the iteration number; and *φ* is a random real number. The fitness between the new food source and the existing food source is compared, and the one with greater fitness is retained. The selection can be defined as

(4) $$x_{i}\left(t+1\right)=\left\{ \begin{array}{ll} v_{i}\left(t+1\right) & if\;fit\left(v_{i}\left(t+1\right)\right)\leq fit\left(x_{i}\left(t\right)\right)\\ x_{i}\left(t\right) & otherwise \end{array}\right.$$

where *fit*() is the fitness function.

At the onlooker bee stage, each onlooker bee makes a selection according to the information of the food source. The probability that an onlooker bee chooses a food source can be calculated by

(5) $$p_{i}=\frac{fit\left(x_{i}\left(t\right)\right)}{\sum_{i=1}^{M}fit\left(x_{i}\left(t\right)\right)}$$

It is clear from Equation (5) that the solution with a greater fitness has a higher probability of being chosen by an onlooker bee. If the onlooker bee has selected a food source, it generates a new solution by Equation (3) and evaluates the fitness by Equation (4).

At the scout bee stage, if the employed bee fails to improve the quality of the solution after a given number of attempts *limit* are achieved, the employed bee becomes a scout bee and the solution it owns is abandoned. The number of employed bees and the number of onlooker bees each account for half of the population size, and the number of scout bees is selected as one.

**Figure 5:**
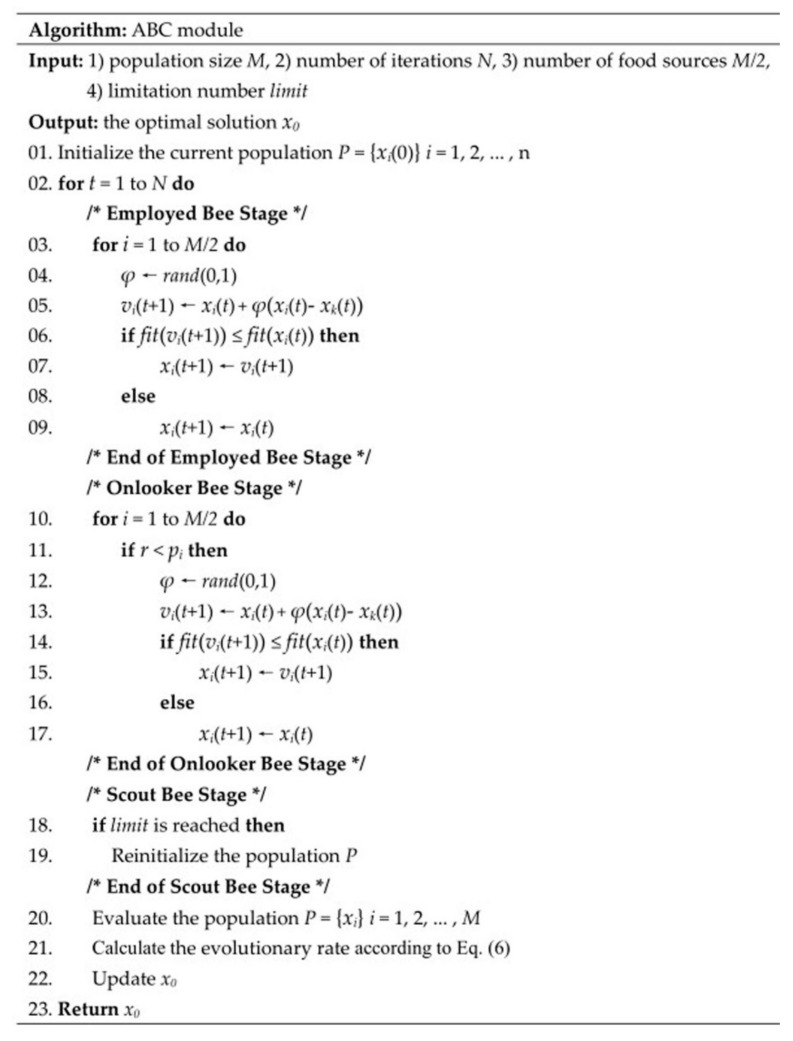
Pseudocode of ABC module.

Subsequently, the evolutionary rate of ABC module is calculated, and the equation is given below:

(6) $$v=\sum_{i=1}^{N}\frac{fit\left(x_{i}\left(t\right)\right)-fit\left(x_{i}\left(t-1\right)\right)}{fit\left(x_{i}\left(t\right)\right)}$$

where *fit*(*x_i_*) is the fitness of an individual *x_i_* at iteration *t*. *fit*(*x_i_*(*t*))-*fit*((*t*-1)) represents the difference between the fitness of the current iteration and that of the previous iteration, and the difference is called evolution velocity. An iteration with a higher evolutionary rate is considered to be more accurate in the search direction of the algorithm. The accuracy of the search direction affects the possibility of finding a better solution in subsequent iterations. Figure 5 gives the pseudocode of ABC module.

### 4.3. DE Module

DE [35] is an efficient global optimization algorithm that simulates biological evolution. Each individual in a population corresponds to a possible solution, and the swarm intelligence generated by mutual cooperation and competition between individuals guides the direction of optimization search. The algorithm starts with a random initial population and gets the optimal solution by iterative mutation, crossover and selection.

A mutated individual is generated by

(7) $$v_{i}\left(t+1\right)=x_{r1}\left(t\right)+F\left(x_{r2}\left(t\right)-x_{r3}\left(t\right)\right)$$

where *x_r_*_1_, *x_r_*_2_, and *x_r_*_3_ are three individuals randomly selected from the previous generation; *t* denotes the iteration number; and *F* is the scalar number. The vector difference between *x_r_*_1_ and *x_r_*_2_ is calculated, and then the vector difference is multiplied by *F* and added to the individual *x_r_*_3_ to be mutated.

A test individual is obtained by crossing the mutant individual and the predetermined target individual. The crossover operation is described as

(8) $$u_{i}\left(t+1\right)=\left\{ \begin{array}{ll} v_{i}\left(t+1\right) & if\;rand\left(0,1\right)<CR\\ x_{i}\left(t\right) & otherwise \end{array}\right.$$

where *rand*(0,1) generates a random number from 0 to 1; and *CR* is the probability of crossover, and it is a constant between 0 and 1.

**Figure 6:**
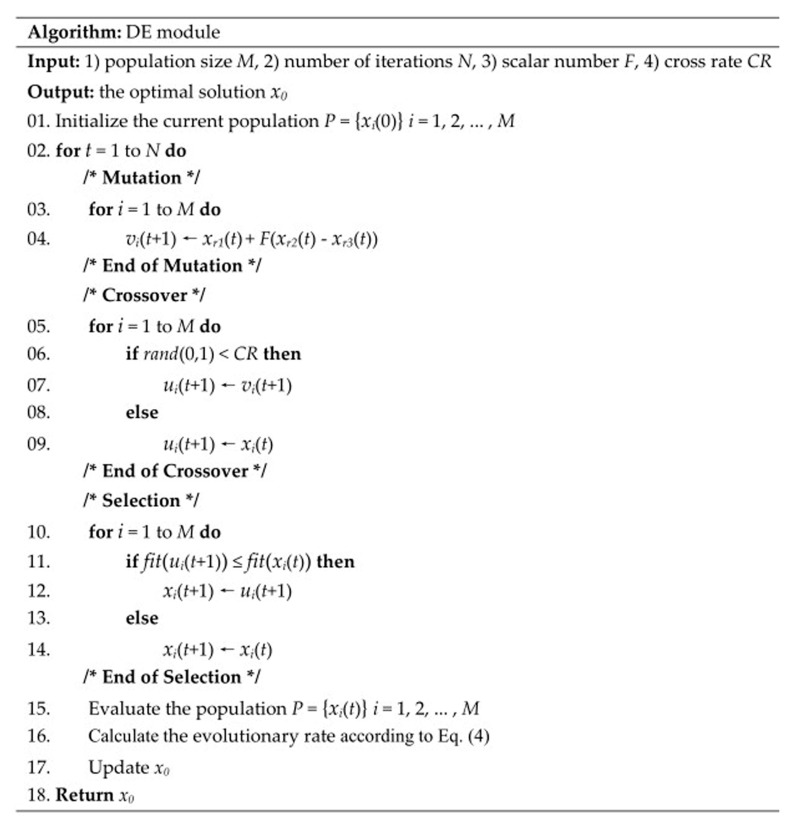
Pseudocode of DE module.

The next generation of individuals is selected by

(9) $$x_{i}\left(t+1\right)=\left\{ \begin{array}{ll} u_{i}\left(t+1\right) & if\;fit\left(u_{i}\left(t+1\right)\right)\;\leq fit\left(x_{i}\left(t\right)\right)\\ x_{i}\left(t\right) & otherwise \end{array}\right.$$

where *fit*() is the fitness function, which generally takes the objective function to be optimized as the fitness function. If the fitness of the test individual is better than that of the current individual, the old individual is replaced by the new individual in the next iteration, otherwise the old individual is still preserved. After executing the three basic operations of DE, the evolution rate is calculated according to Equation (6). The pseudocode of DE module is shown in Figure 6.

### 4.4. APP Module

The population is randomly divided into two different subpopulations. The number of individuals in Subpopulation 1 *P1* and Subpopulation 2 *P2* is *M*_1_ = *M* × *PR* and *M*_2_ = *M* − *M*_1_, where *M* is the total number of the population; and *PR* is the proportion of individuals in Subpopulation 1 called partition rate, which is set to 0.5 as the initial value. After the partition, the individuals of Subpopulation 1 evolve according to ABC module and the individuals of Subpopulation 2 evolve according to DE module.

When an evolution is completed, the emergence probability of new elite individuals in each subpopulation is called elite probability, and the elite probability is expressed according to Equation (10).

(10) $$O_{Z}=\frac{\sum_{j=1}^{E_{Z}}e_{j}\left(t\right)}{\sum_{i=1}^{M_{Z}}x_{i}\left(t\right)}$$

where *M_z_* is the size of a subpopulation; *x_i_* is an individual belongs to the subpopulation at iteration *t*; *E_z_* is the number of elite individuals in the subpopulation; and *e_j_* is an elite individual belonging to the subpopulation at current iteration. *z* is 1 or 2 and represents Subpopulation 1 and Subpopulation 2, respectively. The total size of the population *M* = *M*_1_ + *M*_2_. The total size of the elite individuals *E* = *E*_1_ + *E*_2_. The *E* elite individuals are found at the same time as fitness evaluation, and *E* is typically set to 20% of *M*. The elite probability can reflect the search capability of ABC module and DE module. The more elite individuals there are in this iteration, the greater the probability of continuing to reproduce elite individuals in the next iteration.

Then, the subpopulations are combined and the partition rate *PR* is calculated by Equation (11).

(11) $$PR=\frac{o_{1}\times v_{1}}{o_{1}\times v_{1}+o_{2}\times v_{2}}$$

**Figure 7:**
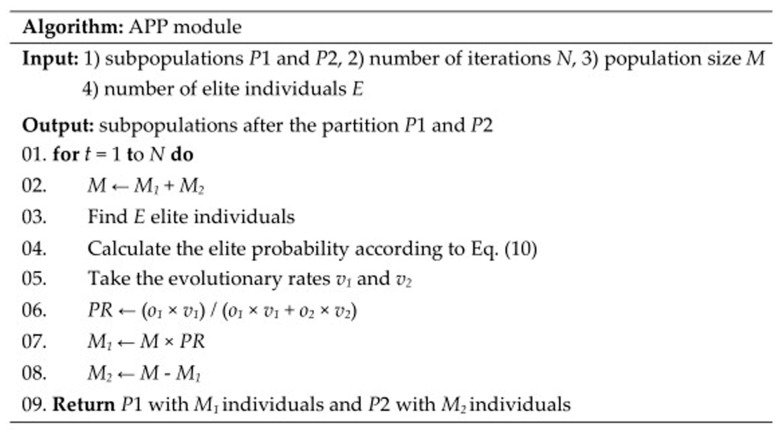
Pseudocode of APP module.

The above partition strategy is called APP. APP adaptively partitions the population and allocates ABC module and DE module in each iteration according to the search situations of these two modules. If the elite probability is simply taken as the basis of the population partition, there will be a situation in which one module accounts for the majority or all of the population and the other module evolves slowly or ceases to evolve because of the lack of individual resources. This is because elite individuals are more likely to reproduce elite individuals. The introduction of the evolutionary rate is a good solution to the situation. The evolutionary rate determines the evolutionary direction of the entire population rather than elite individuals. The evolutionary rate of a module is reduced if the individuals of the module have little impact on the entire population, which can prevent unreasonable partition. The process of partitioning the population is continuously carried out with the iteration of the algorithm until the algorithm reaches a specific terminating condition. Figure 7 gives the pseudocode of APP module.

### 4.5. Hybrid Search of ADHDOCK

ADHDOCK is a hybrid algorithm consisting of ABC module, DE module, and APP module for solving protein–ligand docking problems. In ABC module, the population in the module is evolved according to ABC algorithm. ABC is a specific application of swarm intelligence, and it has a faster convergence rate. Through the local optimization behavior of each artificial bee, the global optimal solution is eventually revealed in the population. In DE module, the population in the module is evolved according to DE algorithm. DE simulates the process of biological evolution, and the individuals who adapt to the environment are preserved after repeated iterations. Compared with GA, the global search strategy based on population is the same, but the complexity of genetic operation of DE is reduced by the simple mutation operation based on difference and the survival strategy of one-to-one competition. ABC and DE have applications in the fields of function optimization, data mining, pattern recognition and so on. The main drawback of these two algorithms is the high probability of falling into local optima, which leads to premature convergence of the algorithms and the high energy values obtained for protein–ligand docking. The reason for the shortcoming is that each algorithm uses a single search strategy.

ADHDOCK applies APP module to combine ABC module and DE module effectively. APP module can reasonably allocate the computational resources of the population in each iteration process. In the initialization of ADHDOCK, the population is partitioned into two subpopulations with the same number of individuals, and the two subpopulations are allocated to ABC module and DE module. After an iterative process of ABC and DE, the two subpopulations are combined. The combined population is repartitioned and then reallocated according to APP module. If one module performs well in this iteration, the module will get more individuals than the other module. The specific usage of APP module is described in the previous chapters. The above process is repeated until a predefined termination condition is reached. If the number of individuals in one module is increasing with the iteration of the algorithm, it can be considered that the module does not fall into local optima. ADHDOCK adopts the search strategies of ABC and DE, and the double search strategy of the novel algorithm can reduce the probability of falling into local optima.

## 5. Conclusions

Protein–ligand docking method has a pivotal part in the field of drug research and provides an effective tool for the discovery and optimization of leading compounds. Studying the interaction between small molecules and protein macromolecules and identifying the target of small molecules in the organisms can help to find a new breakthrough for the development of new drugs. The article presents ADHDOCK, which combines ABC module, ED module, and APP module to solve the protein–ligand docking problem. APP module is responsible for partitioning the population so that computer resources can be used more reasonably. ABC module and ED module execute in parallel under the guidance of APP module, their search capability is maximized. To demonstrate the advantages of ADHDOCK, we also compared the performance of the novel algorithm with that of ABC, DE, LGA, HIGA and SODOCK. Our results indicate that ADHDOCK is superior to the other tested algorithms for the docking problem, in terms of docked energy and RMSD, success rate, convergence performance, data distribution, and hypothesis test.

## Figures and Tables

**Table 1:** Parameters of six tested algorithms.

**ADHDOCK**	
Number of food sources	50
Number of limitation	100
Crossover rate	0.80
Scalar number	0.90
Initial partition rate	0.50
**ABC**	
Number of food sources	50
Number of limitation	100
**DE**	
Crossover rate	0.80
Scalar number	0.90
**LGA**	
Mutation rate	0.02
Crossover rate	0.80
Maximal iterations of local search	300
**HIGA**	
Mutation rate	0.02
Crossover rate	0.80
Maximal iterations of local search	300
Number of elitists	5
equilibrium factor	0.60
**SODOCK**	
Number of immediate neighbors	4
Cognitive weight	2.00
Social weight	2.00
Maximal iterations of local search	300

**Table 2:** root-mean-square deviation (RMSD) results of the first predicted conformations.

Algorithm	Success Case	Average RMSD (All Cases)	Average RMSD (RMSD < 2.0 Å)
ABC	32	3.28 ± 1.32	1.84 ± 0.42
DE	35	3.21 ± 1.37	1.82 ± 0.42
LGA	34	2.55 ± 1.28	1.68 ± 0.40
HIGA	42	1.87 ± 0.99	1.36 ± 0.51
SODOCK	37	2.92 ± 1.08	1.77 ± 0.39
ADHDOCK	46	1.68 ± 0.89	1.19 ± 0.33

**Table 3:** The lowest energy and the smallest RMSD of the best predicted conformations.

PDB	Tor	ADHDOCK		ABC		DE		LGA		HIGA		SODOCK	
		Energy	RMSD	Energy	RMSD	Energy	RMSD	Energy	RMSD	Energy	RMSD	Energy	RMSD
3ptb	0	**−13.25**	**1.65**	−10.95	1.97	−11.23	1.80	−11.53	1.92	−12.22	1.95	−11.57	2.00
1hdy	0	**−10.40**	**0.80**	−8.80	1.47	−8.24	1.05	−8.70	1.78	−9.17	0.98	−9.22	1.49
1aha	1	**−18.20**	0.82	−13.95	1.85	−15.24	1.22	−16.10	**0.45**	−16.15	0.90	−14.95	1.44
1dbb	1	**−12.38**	**0.35**	−11.88	0.88	−11.29	0.55	−11.00	0.72	−11.17	0.80	−11.76	0.88
1mrg	1	**−8.55**	**0.30**	−7.85	0.85	−7.48	1.25	−6.16	0.40	−7.52	0.33	−8.14	1.20
1ulb	1	**−7.50**	0.72	−5.36	0.80	−5.20	0.40	−6.28	0.74	−7.07	**0.35**	−6.75	0.50
1tnl	2	**−9.49**	**0.36**	−5.80	0.68	−6.28	0.52	−6.83	0.73	−8.08	0.62	−6.78	0.88
2phh	2	**−9.21**	0.54	−6.98	1.30	−6.95	1.15	−7.54	0.55	−8.20	0.65	−8.16	**0.34**
3hvt	2	−17.59	0.55	−15.95	0.68	−15.29	0.47	−17.22	**0.33**	**−18.19**	0.45	−16.78	0.58
1phg	3	**−10.28**	**0.38**	−7.95	1.67	−7.90	1.33	−8.56	0.80	−9.58	0.60	−9.15	1.34
2cht	3	**−10.37**	1.55	−7.87	1.24	−8.16	1.16	−8.89	**0.95**	−9.10	1.34	−8.77	1.33
2ctc	3	**−9.25**	**0.78**	−6.40	1.66	−6.70	1.67	−7.70	0.89	−8.90	0.80	−8.52	1.21
4cts	3	**−9.98**	**0.55**	−6.94	0.95	−6.79	0.68	−7.61	0.75	−8.64	0.48	−9.10	1.20
1abe	4	**−10.10**	**0.39**	−7.99	0.95	−8.15	1.03	−8.75	0.60	−9.44	0.75	−8.73	0.80
1hsl	4	**−15.15**	**0.56**	−11.25	1.36	−11.97	1.60	−12.10	0.56	−13.17	0.66	−12.90	1.23
2mcp	4	**−10.35**	**1.05**	−7.85	1.64	−8.10	1.10	−8.22	1.33	−9.35	1.15	−7.72	1.42
1stp	5	**−16.10**	**0.35**	−13.20	1.58	−13.13	0.92	−13.37	1.65	−13.90	0.85	−13.52	1.00
1tni	5	**−9.12**	0.74	−6.82	1.25	−6.79	**0.67**	−8.02	1.65	−8.61	0.90	−7.56	1.34
2lgs	5	**−9.23**	**0.71**	−7.25	1.10	−7.11	0.76	−7.30	0.77	−7.83	1.22	−7.18	1.50
1acm	6	**−11.61**	**0.30**	−9.95	0.33	−9.28	0.40	−10.10	0.37	−10.87	0.33	−10.11	0.45
2cgr	6	**−18.80**	**0.70**	−14.25	0.97	−14.14	0.80	−16.00	0.76	−17.80	0.75	−15.74	0.77
6rnt	6	−9.32	0.55	−8.95	1.45	−8.90	1.65	−9.13	0.70	**−9.62**	**0.50**	−9.12	1.95
1lst	7	**−16.13**	**0.36**	−12.22	0.96	−12.43	0.95	−13.75	0.55	−15.22	0.46	−14.72	0.66
2cmd	7	**−15.14**	**0.42**	−12.70	0.65	−12.42	0.62	−12.26	0.78	−14.05	0.82	−13.28	0.80
4dfr	7	**−13.12**	**1.04**	−10.21	1.97	−10.15	1.20	−11.44	1.23	−12.82	1.56	−11.74	1.67
1ett	8	**−14.90**	**1.20**	−12.75	1.65	−12.40	1.70	−13.89	1.38	−13.94	1.40	−12.08	1.54
1tka	8	**−14.02**	**0.88**	−10.33	1.17	−9.89	1.20	−10.23	0.98	−11.60	1.02	−10.25	1.15
8gch	8	**−14.55**	**0.70**	−10.85	0.82	−11.30	1.15	−11.88	1.72	−12.55	1.66	−11.29	0.98
1hri	9	**−12.03**	**1.13**	−10.13	1.67	−9.98	1.56	−10.21	1.87	−11.02	1.18	−10.31	1.68
1trk	9	**−14.50**	0.80	−11.25	0.65	−11.35	0.62	−11.44	0.65	−13.05	**0.50**	−11.49	0.60
2sim	9	**−18.25**	**0.90**	−15.93	1.10	−15.50	1.06	−15.61	0.95	−16.24	1.08	−15.05	1.06
1eap	10	−14.05	1.25	−12.85	1.21	−12.18	1.30	−13.08	1.27	**−14.55**	**0.98**	−13.77	1.10
1fkg	10	**−17.51**	**1.13**	−15.15	1.20	−15.36	1.22	−15.47	1.36	−16.26	1.35	−15.08	1.38
1hvr	10	**−33.40**	**0.55**	−28.65	0.85	−29.38	0.78	−30.85	0.62	−31.50	0.80	−29.29	0.68
1lna	10	**−15.62**	**1.10**	−13.85	1.82	−13.28	1.67	−13.50	1.75	−15.19	1.29	−13.82	1.22
1nco	11	−21.70	0.93	−20.54	0.77	−20.85	0.82	−21.20	0.65	**−22.75**	**0.55**	−20.60	0.92
4hmg	11	**−10.51**	**1.13**	−9.95	1.60	−10.00	1.28	−10.09	1.70	−10.21	1.65	−10.08	1.36
1bbp	12	**−26.90**	**0.45**	−24.48	0.65	−23.38	0.78	−23.56	0.52	−25.10	0.67	−24.15	0.72
1cdg	12	**−8.95**	**1.05**	−7.13	1.12	−7.72	1.17	−8.22	1.94	−8.90	1.65	−8.45	1.80
1rds	12	**−18.11**	0.75	−16.34	0.92	−15.93	0.86	−16.24	0.80	−17.95	0.77	−16.03	**0.67**
1htf	13	**−22.77**	**1.02**	−19.80	1.80	−20.12	1.48	−20.69	1.33	−21.17	1.20	−21.79	1.42
1glq	14	**−9.83**	**1.15**	−9.23	1.58	−8.53	1.29	−9.27	1.87	−9.65	1.25	−8.83	1.90
1hpv	14	−16.72	1.96	−15.67	1.91	−15.11	1.92	−15.48	1.88	**−17.29**	**1.60**	−15.68	1.75
1qbt	14	**−26.75**	**0.80**	−22.69	1.29	−22.93	1.27	−24.20	1.09	−25.20	0.88	−24.63	1.04
1lic	15	−12.77	**0.85**	−10.01	1.36	−9.80	1.54	−12.17	1.80	**−13.03**	0.96	−12.55	1.08
1tmn	15	**−11.13**	0.90	−9.58	**0.65**	−9.97	1.18	−10.11	1.20	−10.71	0.95	−10.62	1.95
4phv	15	**−22.44**	1.38	−15.62	1.44	−16.08	1.53	−19.18	1.26	−19.89	**0.45**	−21.78	0.90
1epo	17	**−20.33**	**0.80**	−16.07	1.77	−17.18	1.62	−16.80	1.67	−19.13	1.23	−17.65	0.93
1aaq	20	**−23.10**	1.10	−15.55	1.20	−16.60	1.75	−17.44	1.70	−20.66	**1.05**	−19.80	1.34
1hiv	23	**−25.60**	**0.55**	−15.45	1.06	−16.20	0.73	−17.95	1.73	−21.25	1.21	−19.74	1.55

**Table 4:** The lowest energy and the smallest RMSD of the best predicted conformations.

PDB	ABC	DE	LGA	HIGA	SODOCK
3ptb	4.49 × 10^−8^	3.17 × 10^−4^	2.15 × 10^−3^	7.12 × 10^−3^	3.32 × 10^−4^
1hdy	1.18 × 10^−4^	2.39 × 10^−9^	3.04 × 10^−6^	2.50 × 10^−3^	1.02 × 10^−3^
1aha	6.41 × 10^−11^	4.13 × 10^−7^	5.14 × 10^−5^	3.76 × 10^−4^	4.31 × 10^−10^
1dbb	2.27 × 10^−3^	1.03 × 10^−3^	4.15 × 10^−4^	3.25 × 10^−4^	1.43 × 10^−3^
1mrg	2.19 × 10^−4^	6.37 × 10^−5^	2.43 × 10^−8^	4.57 × 10^−4^	1.42 × 10^−1^
1ulb	3.91 × 10^−7^	2.31 × 10^−8^	8.15 × 10^−4^	4.35 × 10^−3^	2.82 × 10^−4^
1tnl	4.93 × 10^−10^	3.86 × 10^−9^	2.62 × 10^−8^	2.14 × 10^−5^	5.90 × 10^−8^
2phh	1.76 × 10^−5^	2.59 × 10^−5^	3.98 × 10^−4^	1.45 × 10^−3^	2.08 × 10^−3^
3hvt	8.99 × 10^−6^	7.34 × 10^−7^	4.12 × 10^−2^	9.98 × 10^−1^	3.06 × 10^−8^
1phg	3.77 × 10^−6^	6.33 × 10^−6^	3.37 × 10^−4^	1.51 × 10^−1^	9.12 × 10^−2^
2cht	1.74 × 10^−8^	2.78 × 10^−7^	4.09 × 10^−5^	2.74 × 10^−4^	6.32 × 10^−5^
2ctc	4.86 × 10^−8^	3.96 × 10^−8^	9.25 × 10^−4^	7.46 × 10^−3^	3.74 × 10^−4^
4cts	3.14 × 10^−8^	1.19 × 10^−9^	3.82 × 10^−6^	2.39 × 10^−4^	7.22 × 10^−3^
1abe	3.38 × 10^−12^	4.31 × 10^−10^	2.35 × 10^−6^	3.55 × 10^−3^	2.04 × 10^−6^
1hsl	1.19 × 10^−10^	3.08 × 10^−8^	1.05 × 10^−6^	5.32 × 10^−4^	3.91 × 10^−5^
2mcp	4.15 × 10^−9^	9.30 × 10^−6^	3.21 × 10^−6^	2.86 × 10^−4^	8.82 × 10^−9^
1stp	2.42 × 10^−7^	1.11 × 10^−7^	8.25 × 10^−6^	3.32 × 10^−5^	3.45 × 10^−6^
1tni	3.14 × 10^−8^	1.43 × 10^−8^	3.71 × 10^−5^	8.46 × 10^−3^	1.58 × 10^−5^
2lgs	2.37 × 10^−5^	1.33 × 10^−9^	2.54 × 10^−8^	4.52 × 10^−5^	2.28 × 10^−4^
1acm	1.42 × 10^−8^	2.12 × 10^−9^	5.18 × 10^−4^	3.15 × 10^−2^	4.12 × 10^−4^
2cgr	3.39 × 10^−7^	1.07 × 10^−9^	3.95 × 10^−4^	2.14 × 10^−2^	7.38 × 10^−4^
6rnt	3.51 × 10^−4^	4.52 × 10^−4^	2.49 × 10^−1^	9.82 × 10^−1^	1.01 × 10^−1^
1lst	1.78 × 10^−10^	2.91 × 10^−8^	3.53 × 10^−5^	5.54 × 10^−3^	2.32 × 10^−4^
2cmd	4.58 × 10^−7^	9.12 × 10^−7^	2.38 × 10^−8^	1.13 × 10^−3^	3.35 × 10^−5^
4dfr	1.26 × 10^−8^	1.09 × 10^−9^	7.74 × 10^−4^	2.52 × 10^−3^	1.34 × 10^−4^
1ett	2.29 × 10^−6^	8.17 × 10^−7^	1.16 × 10^−4^	7.70 × 10^−3^	1.02 × 10^−8^
1tka	4.75 × 10^−8^	3.92 × 10^−10^	2.97 × 10^−7^	3.63 × 10^−5^	2.18 × 10^−7^
8gch	2.48 × 10^−6^	8.19 × 10^−5^	4.08 × 10^−5^	1.40 × 10^−4^	9.03 × 10^−5^
1hri	4.12 × 10^−4^	3.88 × 10^−5^	6.11 × 10^−2^	2.54 × 10^−1^	8.42 × 10^−2^
1trk	1.61 × 10^−8^	5.30 × 10^−7^	3.76 × 10^−7^	2.96 × 10^−3^	1.18 × 10^−7^
2sim	3.29 × 10^−5^	3.97 × 10^−5^	1.05 × 10^−6^	4.52 × 10^−2^	7.12 × 10^−6^
1eap	1.54 × 10^−4^	2.98 × 10^−6^	3.67 × 10^−3^	8.56 × 10^−1^	1.52 × 10^−1^
1fkg	4.92 × 10^−5^	3.73 × 10^−5^	9.13 × 10^−4^	1.19 × 10^−4^	9.62 × 10^−6^
1hvr	1.01 × 10^−7^	2.92 × 10^−5^	1.93 × 10^−4^	8.52 × 10^−3^	8.32 × 10^−5^
1lna	2.29 × 10^−5^	5.37 × 10^−6^	9.10 × 10^−5^	6.13 × 10^−3^	3.40 × 10^−5^
1nco	1.02 × 10^−2^	6.13 × 10^−2^	2.24 × 10^−1^	8.12 × 10^−1^	5.46 × 10^−2^
4hmg	6.15 × 10^−6^	2.23 × 10^−5^	8.75 × 10^−4^	1.40 × 10^−4^	1.07 × 10^−5^
1bbp	1.07 × 10^−5^	8.13 × 10^−7^	2.82 × 10^−7^	4.94 × 10^−3^	2.85 × 10^−5^
1cdg	8.32 × 10^−6^	1.02 × 10^−6^	7.21 × 10^−4^	7.16 × 10^−2^	1.14 × 10^−4^
1rds	3.91 × 10^−4^	4.27 × 10^−7^	6.15 × 10^−4^	6.26 × 10^−2^	1.08 × 10^−5^
1htf	2.24 × 10^−7^	6.27 × 10^−6^	1.80 × 10^−6^	3.42 × 10^−4^	1.17 × 10^−4^
1glq	7.59 × 10^−5^	8.18 × 10^−8^	2.60 × 10^−5^	1.51 × 10^−4^	3.58 × 10^−7^
1hpv	7.12 × 10^−2^	3.92 × 10^−4^	6.15 × 10^−2^	8.15 × 10^−1^	9.33 × 10^−2^
1qbt	8.04 × 10^−9^	2.71 × 10^−9^	1.16 × 10^−6^	2.12 × 10^−4^	5.72 × 10^−4^
1lic	2.43 × 10^−7^	6.39 × 10^−7^	8.18 × 10^−3^	6.88 × 10^−1^	5.89 × 10^−2^
1tmn	2.06 × 10^−7^	6.18 × 10^−7^	2.93 × 10^−6^	3.52 × 10^−4^	9.86 × 10^−4^
4phv	1.35 × 10^−11^	5.13 × 10^−9^	4.75 × 10^−6^	6.56 × 10^−6^	1.12 × 10^−4^
1epo	7.87 × 10^−10^	1.03 × 10^−6^	3.42 × 10^−8^	1.04 × 10^−3^	2.77 × 10^−6^
1aaq	1.02 × 10^−10^	5.52 × 10^−8^	1.24 × 10^−7^	3.18 × 10^−5^	6.19 × 10^−7^
1hiv	8.91 × 10^−9^	4.43 × 10^−8^	6.88 × 10^−7^	2.86 × 10^−4^	1.58 × 10^−6^

## Abbreviations

ADHDOCK	an efficient ABC_DE_based hybrid algorithm for protein–ligand docking
ABC	artificial bee colony
DE	differential evolution
HIGA	running history information guided genetic algorithm
PSO	particle swarm optimization
SODOCK	swarm optimization for highly flexible protein–ligand docking
APP	adaptive population partition
CADD	computer aided drug design
SA	simulated annealing
GA	genetic algorithm
LGA	Lamarckian genetic algorithm
RMSD	Root-mean-square deviation
